# Atypically larger variability of resource allocation accounts for visual working memory deficits in schizophrenia

**DOI:** 10.1371/journal.pcbi.1009544

**Published:** 2021-11-08

**Authors:** Yi-Jie Zhao, Tianye Ma, Li Zhang, Xuemei Ran, Ru-Yuan Zhang, Yixuan Ku

**Affiliations:** 1 Center for Brain and Mental Well-being, Department of Psychology, Sun Yat-sen University, Guangzhou, China; 2 Peng Cheng Laboratory, Shenzhen, China; 3 Institute of Science and Technology for Brain-Inspired Intelligence, Fudan University, Shanghai, China; 4 School of Psychology and Cognitive Science, East China Normal University, Shanghai, China; 5 Shanghai Changning Mental Health Center, Shanghai, China; 6 Shanghai Mental Health Center, Shanghai Jiao Tong University School of Medicine, Shanghai, China; 7 Institute of Psychology and Behavioral Science, Antai College of Economics and Management, Shanghai Jiao Tong University, Shanghai, China; Stockholm University, SWEDEN

## Abstract

Working memory (WM) deficits have been widely documented in schizophrenia (SZ), and almost all existing studies attributed the deficits to decreased capacity as compared to healthy control (HC) subjects. Recent developments in WM research suggest that other components, such as precision, also mediate behavioral performance. It remains unclear how different WM components jointly contribute to deficits in schizophrenia. We measured the performance of 60 SZ (31 females) and 61 HC (29 females) in a classical delay-estimation visual working memory (VWM) task and evaluated several influential computational models proposed in basic science of VWM to disentangle the effect of various memory components. We show that the model assuming variable precision (VP) across items and trials is the best model to explain the performance of both groups. According to the VP model, SZ exhibited abnormally larger variability of allocating memory resources rather than resources or capacity *per se*. Finally, individual differences in the resource allocation variability predicted variation of symptom severity in SZ, highlighting its functional relevance to schizophrenic pathology. This finding was further verified using distinct visual features and subject cohorts. These results provide an alternative view instead of the widely accepted decreased-capacity theory and highlight the key role of elevated resource allocation variability in generating atypical VWM behavior in schizophrenia. Our findings also shed new light on the utility of Bayesian observer models to characterize mechanisms of mental deficits in clinical neuroscience.

## Author summary

Working memory is a core cognitive function related to a broad range of cognitive domains such as problem-solving, attention, executive control, and IQ. Although working memory deficits have been well-documented in schizophrenia, the underlying mechanisms remain unclear. Conventional working memory theories attribute working memory deficits in schizophrenia to their reduced memory capacity, overlooking the potential roles of other memory components, such as precision. In this study, we take the approach of computational psychiatry and use computational modeling to uncover the major determinants of working memory deficits. We assess working memory performance of a large cohort of participants (60 schizophrenia patients and 61 demographic matched healthy controls) and evaluate multiple mainstream computational models of visual working memory. The variable precision model turns out to be the best model for both groups. We further find that the poorer performance of schizophrenia patients arises from heterogeneous distribution of memory resources when encoding items in memory. This resource allocation variability can also predict symptom severity in schizophrenia. Our study highlights the use of computational models in psychiatric researches.

## Introduction

Schizophrenia is a severe mental disorder accompanied by a range of dysfunctions in perceptual and cognitive behavior, among which working memory deficits are considered as one of the core behavioral markers [1–4]. As a key function to temporally store and manipulate information in order to guide appropriate behavior, working memory has been shown to link with a broad range of other cognitive domains, including perception, attention, problem-solving, and executive control [5–8]. Dysfunctions in working memory therefore might cascade into multiple mental processes, causing a wide spectrum of negative consequences [2,3,9].

A well-established finding in lab-based experiments is that people with schizophrenia (SZ) exhibit worse performance than healthy control (HC) participants in visual working memory (VWM) tasks [2]. This phenomenon has long been attributed to decreased VWM capacity in SZ [2,10,11]. The theory of decreased capacity was supported by the previous studies that employed various VWM or other WM tasks, including the ‘span’ tasks (e.g., digit span, spatial span, verbal span) [12,13], the N-back task [14–16], the delayed-response task [17–19], the change detection task [20–24], and the delay-estimation task [11,25,26]. Other reasons that can account for worse WM performance mainly include attention and executive control. SZ patients are more likely to be distracted by task-irrelevant distractors [26–28] and environmental stimuli [29]. This attentional deficit may be related to executive functions that have overlapped cognitive mechanisms and neural networks with working memory [13,30–32]. However, although there are various theories debating the cause of WM deficits in SZ, decreased memory capacity may be the most robust factor that have been proved by many studies.

Besides capacity, researchers have increasingly recognized memory *precision* as another pivotal determinant of VWM performance [33]. Precision reflects the amount of memory resources assigned to individual items—a larger amount of resources assigned to an item results in higher memory precision of that item. At the neural level, low perceptual precision might arise from either the intrinsic noise in neural processing [34–36] or the fluctuations of cognitive factors (e.g., arousal, attention) [36,37]. Atypically increased variability in both behavioral and neural responses has been discovered in patients with mental diseases such as autism spectrum disorder [38,39], dyslexia [40], and attention-deficit/hyperactivity disorder [41]. These theoretical and empirical studies raise the possibility that SZ might have impaired memory precision rather than diminished memory capacity—that is, SZ patients might be able to remember an equal number of items (i.e., comparable capacity) as HC but SZ generally process and maintain items in a less precise manner. Only a few studies have attempted to simultaneously quantify memory capacity and precision in schizophrenic or schizotypy participants, and the results did not reach a consensus [11,25].

Despite the confound of different VWM components as the possible pathological cause, it is unclear whether SZ and HC employ the same computational strategies (i.e., process model) in VWM. Most prior studies only used one model and implicitly assumed that model is the best one for both SZ and HC. But without systematic model comparisons, model optimality cannot be firmly warranted. Conclusions might be biased by the choice of a particular process model. Given that several influential models have been proposed to explain VWM behavior of normal people, it remains unclear which one is the best for SZ. SZ may possess a best-fitting model different from that for normal subjects, indicating that SZ undergo a different computational process when completing VWM tasks. Alternatively, SZ and HC may share the same best model and only differ in some model parameters. These possibilities should be thoroughly tested via transparent model and parameter comparison.

**Fig 1:**
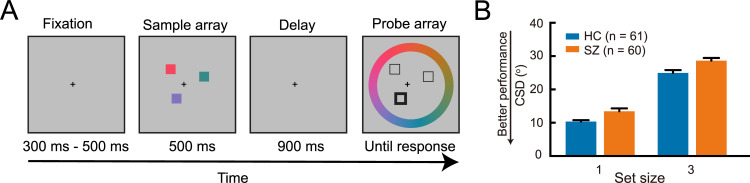
Schematics of the color delay-estimation experiment and behavior results. *A*. Experiment procedure. Participants are instructed to first memorize the colors of all squares (i.e., set size = 3 in this example trial) on the screen, and after a 900ms delay choose the color of the probed square (the one in the left lower visual field in this example) on a color wheel. *B*. Behavior results. Color stimuli can be described in a circular feature space of (0, 360]. Circular standard deviation (CSD) is calculated as the difference between the reported color and the real color of the probe in this standard feature space. SZ show higher CSDs (i.e., worse performance) than HC. All error bars represent SEM across participants.

In the present study, we systematically disentangle the impact of memory capacity and precision, as well as other factors (i.e., variability in allocating resources and variability in choice) in SZ. We measured the performance of SZ and demographically matched HC in a standard VWM delayed-estimation task (Fig 1A). Using the standard task allows us to directly compare our results with those from previous studies [11,42–45]. Most importantly, in contrast to most prior studies, we evaluated and compared several mainstream computational models in the basic research of VWM. This approach allows us to take an unbiased perspective and search a large space of both models and parameters. We also conduct several control experiments and analyses to exclude other confounding factors. We believe that the well-controlled tasks and thorough computational modeling will shed new light on the mechanisms of VWM deficits associated with schizophrenia.

## Results

### Worse VWM performance in SZ in color delay-estimation experiments

The precision of memory in each trial can be quantified as the circular difference (i.e., response error) between the reported color and the true color of the probe. A repeated-measure ANOVA was performed with circular standard deviation (CSD) of response error as the dependent variable, set size (1/3) as the within-subject variable, group as the between-subject variable (Fig 1B). Consistent with findings in previous literature, the participants’ VWM performance was worse with increasing set size levels (F(1, 119) = 641,703, p < 0.001, partial η^2^ = 0.844), and HC performed significantly better than SZ (F(1,119) = 13.651, p < 0.001, partial η^2^ = 0.103). Post hoc analysis showed that SZ performed worse than HC in this task in both set size 1 (p = 0.001, Bonferroni corrected) and set size 3 (p = 0.003, Bonferroni corrected) conditions. The interaction between set size and group was not significant (F(1,119) = 0.229, p = 0.633, partial η^2^ = 0.002). In sum, we replicated the widely documented VWM deficits in schizophrenia patients.

### Variable-precision model accounts for VWM behavior in both HC and SZ

To systematically compare the VWM performance between SZ and HZ, we evaluated several mainstream computational models of VWM briefly introduced as below. Readers may consider skipping the following paragraph to directly reach the result part or go to S1 File for detailed mathematical and intuitive explanations of the models, depending on reading preference.

**Fig 2:**
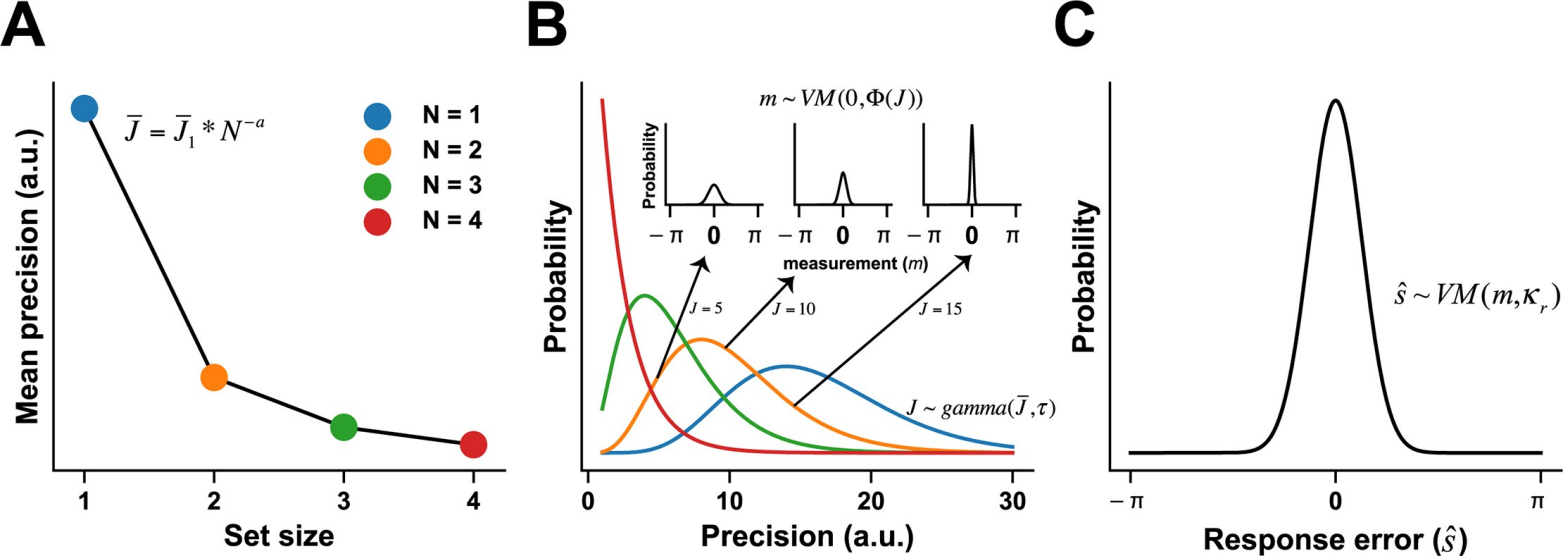
Variable-precision model of VWM. ***A***. Resource decay function. The VP model assumes that the mean resource ($\bar{J}$) for processing a single item declines as a power function of set size *N*, a trend characterized by two free parameters—initial resources ($\bar{J_{1}}$) and decaying exponent (*a*). ***B***. The resources across items or trials follow a gamma distribution with the mean resource ($\bar{J_{1}}$) determined by the resource decay function (***A***) and the resource allocation variability (*τ*). Larger amounts of resources (*J*) indicate higher precision and therefore generate narrower von Mises distributions (three small axes indicating the precision equals to 5, 10 and 15 respectively) of stimulus measurement (*m*). The widths of the von Mises distributions indicate the degree of trial-by-trial sensory uncertainty. ***C***. The eventual behavioral choice given the internal stimulus measurement (*m*) is also uncertain, following a von Mises distribution with the choice variability (*κ*_*r*_) [49]. In the VP model, initial resources ($\bar{J}$), decaying exponent (*a*), resource allocation variability (*τ*) and choice variability (*κ*_*r*_) are four free parameters to estimate (see details in SI and van den Berg *et al*. [50]). All numbers here are only for illustration purposes and not quantitatively related to the model fitting results in this paper. a.u.: arbitrary units.

The first one is the item-limit (IL) model. The IL model assumes no uncertainty in the sensory encoding stage, and that each participant has a fixed memory capacity and a fixed response variability across set size levels [46]. The second one is the mixture (MIX) model, similar to the IL model but assuming response variability is set-size dependent [11,25]. Compared with the MIX model, the slots-plus-averaging (SA) model [42] further elaborates the idea that memory resources manifest as discrete chunks, and these chunks can be flexibly assigned to multiple items. We also explored a modified version of the SA model, dubbed cosSA model, which inherits the idea of discrete memory resources and further assumes that response bias is stimulus-dependent and can be described as empirically derived periodic functions. The fifth one is the equal-precision (EP) model, which is similar to the variable-precision (VP) model (Fig 2) below but assumes that the memory resources are evenly distributed across items and trials [47,48]. The VP model proposes that memory resources are continuous, and the amount of resources assigned to individual items varies across items and trials. Note that the VP model does not include the capacity component thus we also constructed a variable-precision-with-capacity (VPcap) model that not only acknowledges the variable precision mechanisms but also explicitly estimates the capacity of individual participants. Note that the IL, MIX, SA and cosSA, and VPcap models have the parameter of *capacity*, but the EP and VP models do not. Here, capacity is operationally defined as the maximum number of items that can be stored in memory. Some items must be out of memory if set size exceeds capacity, and the participant has to randomly guess the color if the probe is one of these out-of-memory items.

**Fig 3:**
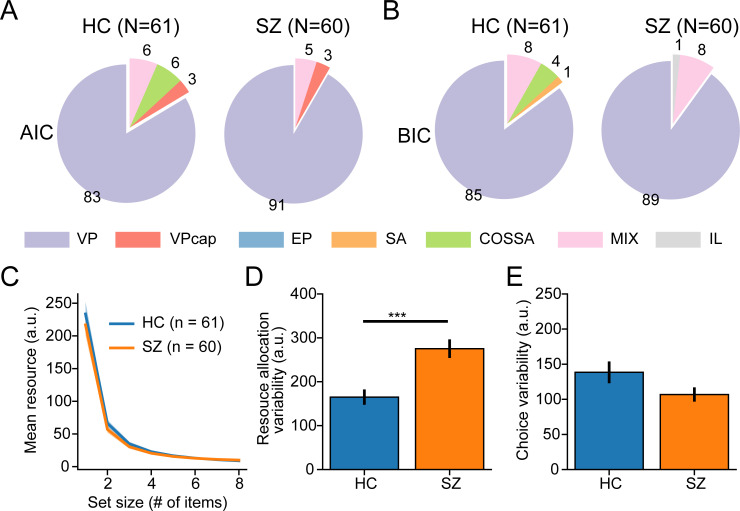
Model fitting results. ***A*** *&* ***B***. Model comparison results. The pie charts illustrate the proportion (i.e., the percent number shown along with each slice) of the participants for whom each model is their best-fitting model. Under both AIC (*A*) and BIC (*B*) model comparison metrics, the VP model is the best-fitting model for the majority of participants in both groups. ***C*** *to* ***E***. Fitted parameters of the VP model. No significant group differences are noted between the two groups in resource decay functions (*C*), and choice variability (*D*). SZ have larger resource allocation variability than HC (*E*). Note that we extrapolate the resource decay function to higher set size levels to visualize the overlapping trend of the two groups. The solid lines represent the averaged resource decay functions across participants. The shaded areas in panels A and all error bars in the other panels represent ±SEM across participants. Significance symbol conventions are ***: p < 0.001. a.u.: arbitrary units.

We compared all seven models using the Akaike information criterion (AIC) and the Bayesian information criterion (BIC) [51,52] in each participant. We found the VP model was the best-fitting model for over 84% of the participants in the HC group according to both metrics (Fig 3A and 3B), replicating previous results in normal people [50,53]. Most importantly, the VP model was also the best-fitting model for over 90% of the participants in the SZ group (Fig 3A and 3B). This result indicates that both groups use the same process model to perform the task.

The superior performance of the VP model indicates the important role of variable precision in VWM processing. It is worth noting that the VP model, as the best-fitting model, does not include a parameter of capacity. Therefore, we cannot conclude that the two groups have the same capacity. But this result highlights the importance of performing systematic model comparisons before the analysis of group differences on model parameters.

### Larger resource allocation variability in SZ

Analyses above have established that HC and SZ employ the qualitatively same process model to complete the VWM task. Their behavioral differences thus should arise from the differences in some parameters in the process model. We next compared the fitted parameters of the VP model across the two groups. We found that the two groups had comparable resource decay functions (Fig 3C, initial resources, t(119) = 0.689, p = 0.492, Cohen’s d = 0.125; decaying exponent, t(119) = 1.065, p = 0.289, Cohen’s d = 0.194), indicating a similar trend of diminished memory resources as increasing set size. SZ, however, had larger variability in allocating resources (Fig 3D, resource allocation variability, t(119) = 4.03, p = 9.87 × 10^−5^, Cohen’s d = 0.733). Furthermore, the VP model explicitly distinguishes the variability in processing items and the variability in exerting a behavioral choice (e.g., motor or decision noise). We found no significant group difference in the choice variability (Fig 3E, t(119) = 1.7034, p = 0.091, Cohen’s d = 0.31), excluding the possibility that the atypical performance of SZ arises from larger variability at the choice stage.

These results suggest that, although the two groups have on average the same amount of memory resources across different set size levels, SZ allocate the resources across items or trials in a more heterogeneous manner, with some items in some trials receiving considerably larger amounts and vice versa in other cases. Given that the two groups only differ in this parameter, unbalanced resource allocation leads to larger trial-by-trial response errors in SZ. We further established the causal link between increased resource allocation variability and larger response errors via computer simulation (see S5 Fig). Note that no explicit reward was used in this task, such behavioral differences may arise from different objectives the two groups attempt to optimize against. Also, in the condition of set size equal to 1 (i.e., only one item is presented), resource allocation cannot vary across items but still vary across trials, leading to worse performance in SZ. We further quantitatively confirmed that increased resource allocation variability indeed leads to larger behavioral response errors (see S5 Fig).

### Resource allocation variability predicts the severity of schizophrenic symptoms

Next, we sought to investigate whether the results from the VP model can predict clinical symptoms. A set of correlational analyses were carried out to link the estimated resource allocation variability to the schizophrenia symptomatology in each patient. BPRS, SANS, and SAPS questionnaires were administered for each patient (Table 1).

**Table 1:** Demographics and clinical information of people with schizophrenia (SZ) and healthy control (HC) participants.

	SZ (N = 60)		HC (N = 61)	
	Mean	SD	Mean	SD
Age	35.67	6.58	33.82	9.90
range	23–48	n/a	21–57	n/a
Female/male	31/29	n/a	29/32	n/a
Inpatient/outpatient	33/27	n/a	n/a	n/a
Subject’s education (in years)	12.03	2.24	15.13	3.70
Paternal education (in years) ^a^	9.89	2.53	9.76	2.95
Maternal education (in years)	9.62	2.91	9.29	3.63
BPRS	27.25	6.27	n/a	n/a
SAPS	5.77	7.02	n/a	n/a
SANS	24.43	11.45	n/a	n/a

^a^ Average of mother’s and father’s years of education

BPRS: Brief Psychiatric Rating Scale [54]; SAPS: Scale for the Assessment of Positive Symptoms [55]; SANS: Scale for the Assessment of Negative Symptoms [56].

**Fig 4:**
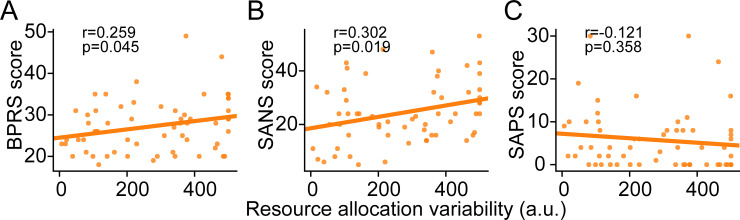
Individual differences in resource allocation variability predict the scores in symptom assessments. In the color experiment, estimated resource allocation variability values in the SZ group significantly correlates with their scores on BPRS (*A*) and SANS (*B*, negative symptoms) but not on SAPS (*C*, positive symptoms). a.u.: arbitrary units.

We noticed that the estimated resource allocation variability values of the SZ participants correlated with their BPRS scores (Fig 4A, r = 0.259, p = 0.045) and SANS scores (Fig 4B, r = 0.302, p = 0.019). No significant correlation was noted on the SAPS scores (Fig 4C, r = -0.121, p = 0.358). These results suggest that resource allocation variability not only is the key factor explaining VWM deficits in SZ but also quantitatively predicts the severity of symptoms, highlighting its promising utility as a behavioral marker in future diagnosis and rehabilitation of schizophrenia.

### Control experiments and analyses

To examine the robustness of our results, we run three additional control experiments or analyses to exclude other confounding factors. Detailed methods and results are in the S2 File.

One possible explanation for the worse VWM performance in SZ is that the deficits are related to their general worse ability of color perception rather than memory. To exclude this possibility, in the control color perception experiment (see details Materials and Methods section), we tested the color perception of the participants in the main experiment. We found that resource allocation variability is still significantly heightened in SZ if individual differences in color perception thresholds are controlled (see details in S2 File).

Another possible explanation for our results is that we only included easy testing conditions (e.g., set size levels 1 and 3) and did not challenge participants’ ceiling performance. Due to relatively low set size levels, the optimal model may be unspecified (i.e., the VP model may not be the best model) or the estimations of capacity may be imprecise. We want to highlight that our results are consistent with the previous studies where higher set size levels were used [50,53]. SZ patients usually cannot bear a long experiment and the high-set-size conditions are too difficult for them, leading to a high drop-out rate. We thus chose the relatively easy experiment setup to ensure the recruitment of a large cohort of participants. Nonetheless, we performed a high-set-size color delay-estimation experiment (see details in Materials and Methods section) on another cohort of normal subjects (N = 62). In this experiment, participants were tested on set size levels 2, 4, and 6. We performed the same analyses and found that the VP model was still the best-fitting model in over 70% of the participants (S3 Fig). Note that this analysis only ensures our setup (set sizes 1/3) is able to identify best-fitting model, providing no additional evidence for any potential difference between SZ and HC.

To further substantiate larger resource allocation variability in SZ, we performed another control experiment. SZ’s visual working memory deficits are ubiquitous across many tasks. To test whether our results here are only specific to color visual working memory, we performed another orientation delay-estimation task where participants were asked to memorize the orientations of a set of bars and reproduce the orientation of a target bar (S4A Fig). This task included three set size levels (2/4/6), and 26 HC and 9 SZ participants were tested on this task. We performed a whole set of analyses as we did in the main experiment and replicated the findings in our main experiment. We found that the VP model was still the best model in both groups (S4D Fig), and resource allocation variability, as the only group difference, was significantly higher in the SZ group (S4E Fig). Most importantly, we again found significant correlations between estimated resource variability and individuals’ negative symptom scores (S4F Fig).

In sum, the main experiment and three additional control experiments together included a total of 149 HC and 69 SZ participants. Converging results from these experiments demonstrate the converging result that the VP model is the best model for both HC and SZ participants. This finding is unlikely due to idiosyncrasies in experimental settings or model fitting.

## Discussion

The mechanisms underlying VWM deficits of schizophrenia have been a matter of debate over the past few years. Abnormally decreased capacity has been widely proposed as the major cause of the deficits in SZ. In the present study, we re-examine this conclusion by comparing the performance of SZ and HC using several mainstream computational models of VWM proposed so far. We first establish that the VP model is the best model to characterize the performance of both groups. We then further evaluate different components in the VP model and find that SZ have larger variability in the memory resources allocated across memoranda and trials. These findings are highly consistent in two independent samples of participants and in two independent behavioral tasks. Furthermore, in two independent experiments, individual differences in resource allocation variability predict variations of patients’ severity of negative symptoms, highlighting its clinical functionality. Taken together, our results propose for the first time that resource allocation variability is the key factor that limits VWM performance in schizophrenia.

A large body of literature has documented that SZ perform poorly in various forms of working memory tasks [2,3,57,58]. The majority of the studies reached the same conclusion that the working memory deficits arise from decreased memory capacity in schizophrenia. However, the definition of *capacity* varies substantially across studies. Many studies directly equated worse performance with decreased capacity without quantitatively demonstrating how capacity modulates performance. For example, memory capacity was defined as the number of digits that can be recalled in the longest strand in digit span tasks [12]. In N-back tasks, capacity was defined as the number of backs corresponding to a certain accuracy level [14–16]. Moreover, the calculation of capacity resembled the d-prime metric in change detection tasks [22–24,46,59]. The majority of these metrics are actually behavioral thresholds or accuracy related to capacity rather than direct quantifications of capacity. This is partly because we lack appropriate computational models for most of those tasks. The VP model is advantageous as it describes the generative process of the delay-estimation task and the change-detection task [50]. Therefore, it allows for disassociating the effect of capacity from other VWM components. Note that here we only consider resource amount, capacity, resource allocation, and choice as the dimensions of modeling. Some studies suggest the existence of “binding error” in VWM [60,61]. Van den Berg et al 2014 [53] explored a large space of model parameters and found that “binding error” only accounts for a small fraction of VWM limitation. As such we did not consider “binding error” in this paper. Also, all computational models we used here are specific to the delay-estimation task. It is possible that the VWM deficits of schizophrenia arise from distinct mechanisms in different tasks. Future studies are needed to test whether larger resource allocation variability can also account for VWM deficits of SZ in other WM tasks.

Only a few studies have quantitatively estimated capacity and precision in schizophrenia. Gold et al [11] employed the same color delay-estimation task as that in our study and estimated individuals’ capacity and precision using the MIX model. Results in that study echoed the decreased-capacity theory. The MIX model, however, does not consider two important factors. First, the model assumes an equal precision across items in memory. Second, the model does not separate the variability for processing stimuli (i.e., sensory uncertainty, *κ* in S1 File Eq. S5) and the variability in exertion of a choice (i.e., choice uncertainty, *κ*_*r*_ in S1 File Eq. S6). This distinction is important since it highlights different types of uncertainty in encoding and decoding stages of VWM. Mathematically, these two types of uncertainty can be distinguished by manipulating set size since theoretically set size only influences the encoding variability but not the choice variability. The issues of the MIX model have been symmetrically addressed in a recent study [62].

Compared with capacity and precision—the two diagnostic features of VWM—resource allocation variability emerges as a new concept in VWM. It refers to the heterogeneity of allocating resources across multiple items and trials. Systematically delineating the neural sources of resource allocation variability is beyond the scope of this paper. We speculate that resource allocation variability could arise from both top-down cognitive fluctuations (e.g., attention) and bottom-up neural variability for several reasons. First, it has been shown that attention and WM are two core components of executive control and tightly linked with each other [31,63]. Second, schizophrenia is known to have deficits in top-down attentional modulation [31,58]. Particularly, recent studies have discovered the phenomenon of spatial hyperfocusing in schizophrenia patients [19,64–66]. If schizophrenia patients overly attend to one item and ignore others in the memory encoding stage, unbalanced resource allocation will likely occur. Third, it has been well-documented that neural variability plays an important role in perceptual and cognitive processing even without top-down modulations [67,68], and abnormal levels of variability have been found in several other mental diseases [38–41]. Also, a recent study found that variable precision is even more likely to be driven by stimulus-specific effects [69]. Note that SZ also exhibit worse performance when only one target is presented. In this condition, resources do not vary across items (i.e., only one item) but vary across trials. Also, we want to emphasize that such variability is not equivalent to attentional lapse. A higher attentional lapse rate may lead to overall fewer resources, a phenomenon we did not observe in this study.

What are the neural mechanisms of this resource allocation variability? Recent neurophysiological studies have proposed that the neural representation of a stimulus may follow a doubly stochastic process [67,68], which suggests that the variability in encoding precision is a consequence of trial-to-trial and item-to-item fluctuations in attentional gain [37,50,70]. A recent study combined functional magnetic resonance imaging and the VP model, showing that the superior intraparietal sulcus (IPS) is the cortical locus that controls the resource allocation [71]. Interestingly, schizophrenia patients have been known to have IPS deficits [72].

The current results also reveal links between resource allocation variability and patients’ negative symptoms, but not positive symptoms (Fig 4). These findings are consistent with previous studies claiming dissociable mechanisms underlying the cluster of negative symptoms versus that of positive symptoms. Cameron and his colleagues have looked into different working memory tasks and showed that deficits in verbal fluency task and visuospatial working memory task are correlated with severity of negative symptoms while no tested tasks are correlated with positive symptoms [73]. Similar results have been found in both spatial [74] and non-spatial [75] delayed response tasks showing worse working memory performance with severer negative symptoms in SZ. A meta-analysis study reviewing various neurocognitive domains has illustrated that intelligence quotient (IQ), reasoning and problem solving, verbal learning and memory, verbal fluency and attention abilities are correlated with negative symptom in SZ but not with positive symptoms. Only the speed of processing is correlated with both negative and positive symptoms [76]. It is notable that although most studies exhibit null correlation between general positive symptoms and working memory performance, one aspect of positive symptoms: disorganization has been shown to be in relation to visuospatial working memory, verbal fluency and set-shifting abilities [73] as well as IQ, reasoning and problem solving and attention [76]. Unfortunately, the scale used in our study doesn’t have the dimension of disorganization, so that we couldn’t do correlational analyses on this symptom dimension. More broadly, a growing collection of evidence suggests that visual perceptual deficits in schizophrenic patients are more likely to link to negative rather than positive symptom severity [77–81]. Negative symptoms in turn might produce improvised social functioning. Humans depend heavily on VWM to interact with multiple agents and complete social tasks. Deficits in distributing processing resources over multiple agents therefore might cause disadvantages in social cognition.

In conclusion, our study proposes a new explanation that the resource allocation variability accounts for the atypical VWM performance in schizophrenia. This view differs from the decreased-capacity theory and provides a new direction for future studies that attempt to promote diagnosis and rehabilitation for schizophrenic patients.

## Materials and methods

### Ethics statement

All experimental protocols were approved by the institutional review board at the East China Normal University. All research was performed in accordance with relevant guidelines and regulations. Informed written consent was obtained from all participants.

#### Color delay-estimation experiment participants

61 HC and 60 SZ participated in the experiment. SZ were clinically (symptom and medication) stable inpatients (N = 33) and outpatients (N = 27) who met DSM-IV criteria [82] for schizophrenia. Patients having a history of any other mental or neurological disorders were excluded. All patients were receiving antipsychotic medication (2 first-generation, 43 second-generation, 15 both). Symptom severity was evaluated by the Brief Psychiatric Rating Scale (BPRS) [54], the Scale for the Assessment of Negative (SANS), and Positive Symptoms (SAPS) [55,56]. HC were recruited by advertisement. All HC had no current diagnosis of axis 1 or 2 disorders as well as no family history of psychosis nor substance abuse or dependence. All participants are right-handed with normal sight and color perception.

The two groups were matched in age (t(119) = 1.58, p = 0.118, Cohen’s d = 0.284), gender (31 females and 29 males) and education level of parents (t(119) = 0.257, p = 0.798, Cohen’s d = 0.047). Inevitably, the SZ had fewer years of education than the HC (t(119) = 5.51, p = 2.09 × 10^−7^, Cohen’s d = 1.00). The detailed demographic information is summarized in Table 1.

#### Stimuli and task

The participants sat 50 cm away from an LCD monitor. All stimuli were generated by Matlab 8.1 and Psychtoolbox 3 [83,84], and then presented on the LCD monitor.

In the color delay-estimation task (Fig 1A), each trial began with a fixation cross presented at center-of-gaze for a duration randomly chosen from a sequence of 300, 350, 400, 450, and 500 ms. The participants shall keep their fixation on the cross throughout the whole experiment. A set of colored squares (set size = 1 or 3) was shown on an invisible circle with 4^o^ radius. Our pilot experiment showed that SZ patients exhibit a high dropout rate if the task is longer than 30 mins or too hard (i.e., set size > 4). We therefore reduced the difficulty of the color task to set size levels 1 and 3. The sample array lasted 500 ms. Each square was 1.5^o^ × 1.5^o^ of visual angle. Their colors were randomly selected from the 180 colors that are equally distributed along the wheel representing the CIE L*a*b color space. The color wheel was centered at (L = 70, a = 20, b = 38) with a radius of 60 in the color space [42]. The sample array then disappeared and was followed by a 900 ms blank period for memory retention. After the delay, an equal number of outlined squares were shown at the same location of each sample array item, with one of them bolded as the probe. In the meantime, a randomly rotated color wheel was shown. The color wheel was 2.1^o^ thick and centered on the monitor with the inner and the outer radius as 7.8^o^ and 9.8^o^ respectively. The participants were asked to choose the remembered color of the probe by clicking a color on the color wheel using a computer mouse. The participants needed to choose the color as precisely as possible and response time was not constrained. The participants completed 2 blocks for the set sizes 1 and 3, respectively. The order of the two blocks was counterbalanced across participants. Each block had 80 trials. The difference between the reported color and the true color of the target was considered as the response error.

## Supporting information

S1 File Computational models of visual working memory and intuitive model explanations. (DOCX)

S2 File Control experiments 1 to 3. (DOCX)

S1 Fig Cartoon illustration of all computational models considered in this study. This figure aims to aid an intuitive understanding of the models. Detailed model explanations are in the section of ***intuitive model explanations***. ***A***. item-limit model; ***B***. MIX model; ***C***. the principle of discrete slots and the SA model; ***D***. cosSA model; ***E***. the principle of continuous resources; ***F***, EP, VP, and VPcap models. See the section of ***intuitive model explanations*** for detailed explanations.
(EPS)

S2 Fig Distributions of response errors across different set size levels in the main color delay-estimation experiment. In both set size levels, the distributions of errors are wider in SZ subjects than those in HC subjects. Note that the standard deviations of these distributions are plotted in Fig 1B.
(EPS)

S3 Fig Model comparisons on the data of 62 HC participants tested in the control experiment 2: high-set-size color delay-estimation task. Note that different from the color task in the main text, these subjects were tested on three set size levels (2/4/6). Model comparison results are consistent with those in the main text: the VP model is the best one when set size levels are increased.
(EPS)

S4 Fig Schematics of the orientation delay-estimation experiment and results. ***A***. Experiment procedure. Participants are instructed to remember the orientations of a set of bars (i.e., set size = 4 in this example trial) and then adjust the orientation of the probe to match the memorized orientation using a mouse. ***B***. Behavior results shown as similar to Fig 1B. Orientation stimuli can be described in a circular feature space of (0, 180]. Circular standard deviation (CSD) is calculated as the difference between the reported orientation and the real orientation of the probe in this standard feature space. SZ show higher CSDs (i.e., worse performance) than HC. All error bars represent SEM across participants. ***C***. distributions of response errors shown as similar to S2 Fig. ***D***. model comparison results. The pie charts illustrate the proportion (i.e., the percent number shown along with each slice) of the participants for whom each model is their best-fitting model. Under both AIC and BIC model comparison metrics, the VP model is the best-fitting model for the majority of participants in both groups. ***E***. Fitted parameters of the VP model. No significant group differences are noted between two groups in resource decay functions (left panel), and choice variability (right panel). SZ have larger resource allocation variability than HC (middle panel). The solid lines represent the averaged resource decay functions across participants. The shaded areas in the left panel and all error bars in the other panels represent ±SEM across participants. Significance symbol conventions are ***: p < 0.001. ***F***. Individual differences in resource allocation variability predict the scores in symptom assessments in the orientation delay-estimation experiment. Resource allocation variability significantly correlates with the PANSS general scores (upper panel), PANSS negative scores (middle panel). This correlation is not significant for PANSS positive scores (bottom panel).
(EPS)

S5 Fig Simulation of the behavioral consequences of increased resource allocation variability. Based on the VP model, we simulate 4000 behavioral responses in each parameter combination (SS: set size, J_1: initial resources, a: decaying exponent, k_r: choice variability). We systematically manipulate resource allocation variability and initial resource, and fix decaying exponent and choice variability to the group average of the fitted parameters of the HC group. Increased resource allocation variability leads to large response errors, indicating that low resource allocation variability is more optimal in this task.
(EPS)

S1 Table Demographics and clinical information of the participants in the orientation delay-estimation task. (DOCX)
