# Supplementary material for: Atypically larger variability of resource allocation accounts for visual working memory deficits in schizophrenia
Source: PLoS Comput Biol. 2021 Nov 8;17(11):e1009544. doi: 10.1371/journal.pcbi.1009544 (PMC8601612; doi:10.1371/journal.pcbi.1009544)
Supplement: S1 File — (DOCX) [file pcbi.1009544.s001.docx]

# S1 File. Computational models of visual working memory and intuitive model explanations

# Computational models of visual working memory

## Variable-precision model

The variable-precision (VP) model has been shown as the state-of-the-art computational model of VWM. Details of the VP model have been documented in several previous studies [1,2] and the model codes are publicly available (<http://www.cns.nyu.edu/malab/resources.html>).

The VP model assumes a resource decaying function describing the decreasing trend of mean memory resource ($\overline{J}$) assigned to individual items as the set size (*N*) increases [3,4]:

$\overline{J}=\overline{J_{1}}*N^{-a}$ , (S1)

where $\overline{J_{1}}$ is the initial resources when only 1 item (*N* = 1) should be memorized and *a* is the decaying exponent. The key component of the VP model is that the memory resources $J$ across items and trials follow a Gamma distribution with the mean $\overline{J}$and the scale parameter $\tau$:

$J\sim Gamma(\overline{J},\tau)$ , (S2)

Intuitively, a larger $\tau$ indicates a more uneven distribution of memory resources across items or trials, with some items in some trials receiving a larger amount of resources while others receive comparative fewer. Note that a larger amount of memory resource produces a higher precision. Thus, we do not explicitly distinguish resource and precision and denote them as *J.* Defining precision as Fisher information [5], precision $J$ can be linked to the variance of the von Mises distribution of sensory measurement:

$J=\kappa\frac{I_{1}\left(\kappa\right)}{I_{0}\left(\kappa\right)}$, (S3)

where $I_{0}$ and $I_{1}$ are modified Bessel functions of the first kind of order 0 and 1 respectively, with the concentration parameter $\kappa$. Eq. S3 specifies a one-on-one mapping between precision $J$ and variance $\kappa$. We can rewrite their relationship as:

$\kappa=\Phi(J)$ , (S4)

where $\Phi$ is the mapping function. The distribution of sensory measurement (*m*) given the input stimulus (*s*) can be written as:

$p(m|s)=\frac{1}{2\pi I_{0}(\kappa)}e^{\kappa\cos(m-s)}\equiv VM(m;s,\kappa)$, (S5)

We further assume that the reported color ($\hat{s}$) by participants also follows a von Mises distribution:

$p(\hat{s}|m)=\frac{1}{2\pi I_{0}(\kappa_{r})}e^{\kappa_{r}\cos(\hat{s}-m)}\equiv VM(\hat{s};m,\kappa_{r})$, (S6)

where $k_{r}$ represents the variability at the choice stage.

Given the four free parameters and stimulus color $s$ in a trial, we can derive the probability of the observed response in a trial by marginalizing over sensory measurement $m$ and variable precision $J$:

，(S7)

Note that in Eq. S7, sensory measurement (*m*) can be analytically eliminated. Since precision $J$is a random variable across items and trials, we sampled it 10000 times from the Gamma distribution with mean $\overline{J}$ and scale parameter $\tau$. Note that van den Berg *et al*. [1] confirmed that 500 samples are enough in the model fitting. We then used all the samples to calculate response probability in each trial.

Taken together, this VP model has four free parameters: $\overline{J_{1}}$, *a*, $\tau$and $\kappa_{r}$.

## Variable-precision-with-capacity model

The variable-precision-with-capacity (VPcap) model inherits all parameters and the structure of the VP model above, except that an additional capacity parameter (*K*) is introduced to estimate the memory capacity of individuals. If the set size *N* is smaller than capacity *K*, the VPcap model is identical to the VP model. If the set size *N* exceeds the capacity *K*, the model assumes that the probe is stored in the VWM with the probability *K*/*N*, and out of memory with the probability 1- *K*/*N*. In the latter case, a participant randomly guesses a color. The response probability therefore can be written as:

$p(\hat{s}|s)=\left\{\begin{matrix} \frac{K}{N}p(\hat{s}|s;\bar{J},\tau)+(1-\frac{K}{N})\frac{1}{2\pi}, & K<=N \\ p(\hat{s}|s;\bar{J},\tau), & K>N \end{matrix}\right.$, (S8)

where $p(\hat{s}|s;\bar{J},\tau)$ is defined in Eq. S7. In essence, the VPcap model is a mixture model of the VP model and a random guessing process when the set size exceeds the participant’s capacity. The VPcap model has five parameters, four as the same in the VP model and the additional capacity parameter (*K*).

## Item-limit model

The item-limit (IL) model assumes no uncertainty in the sensory encoding stage such that the internal sensory measurement *m* is equal to the input stimulus *s*. But there exists choice variability from measurement *m* to the reported color ($\hat{s}$). Such choice variability does not vary across set size levels. The IL model also assumes a fixed capacity *K.* The response probability is:

$p(\hat{s}|s)\equiv p(\hat{s}|m)=\left\{\begin{matrix} \frac{K}{N}VM(\hat{s}|s,\kappa_{r})+(1-\frac{K}{N})\frac{1}{2\pi}, & K<=N \\ VM(\hat{s}|s,\kappa_{r}), & K>N \end{matrix}\right.$ , (S9)

The IL model has two free parameters: choice variability $\kappa_{r}$, and capacity *K*.

## Mixture model

The mixture model (MIX) has been used in previous clinical research [6]. Similar to the IL model, the MIX model only assumes the uncertainty from stimulus *s* to the reported color ($\hat{s}$) and a fixed capacity *K.* The difference is that the uncertainty ($\kappa$) reflects both sensory noise and choice variability, and thus the uncertainty is set-size dependent (each set size has one $\kappa$). The response probability can be written as:

$p\left(\hat{s}|s\right)=\left\{\begin{matrix} \frac{K}{N}VM\left(\hat{s}|s,\kappa_{1/3}\right)+\left(1-\frac{K}{N}\right)\frac{1}{2\pi}, & K\leq N \\ VM\left(s|s,\kappa_{1/3}\right), & K>N \end{matrix}\right.$, (S10)

where and denote the uncertainty for set size 1 and 3, respectively. The MIX model has three parameters: uncertainty levels $\kappa_{1}$ and $\kappa_{3}$, and capacity *K.*

**Slots-plus-averaging model**. The slots-plus-averaging (SA) model was originally proposed in [7] and further elaborated in [1]. Unlike the IL model, the SA model acknowledges the presence of noise in the sensory encoding stage. However, the memory resources are discrete chunks, and a single chunk or multiple chunks can be assigned to one item. For one item, the SA model assumes Eq. S4 still holds as the relationship between the resource assigned to that item and the width of the von Mises distribution:

$\kappa=\Phi(SJ_{s})$ , (S11)

where *S* is the number of chunks and *J_s_* is the resource of one chunk. The SA model also assumes a capacity *K*.

When *N* > *K*, an item should receive either 0 or 1 chunk. Then the allocation should be similar to the IL model. the response distribution should be a mixture of a uniform and a von Mises distributions:

, (S12)

When N ≤ K, some items receive either one or more chunks. Assuming that the resource chunks should be assigned as equally as possible across items, the *S* can be calculated as:

$S=\left\{\begin{matrix} \left\lfloor\frac{K}{N}\right\rfloor, & \text{with probability 1-}\frac{K\mathrm{mod}N}{N} \\ \left\lfloor\frac{K}{N}\right\rfloor+1, & \text{with probability }\frac{K\mathrm{mod}N}{N} \end{matrix}\right.$, (S13)

where $\left\lfloor x\right\rfloor$ represents the *floor* function in Matlab. The corresponding concentration parameter of von Mises distributions can be computed by Eqs. S11&13:

(S14)

The response probability in the SA model can be written as:

, (S15)

The SA model has three free parameters: unit resource *J_s_*, choice variability $\kappa_{r}$, and capacity *K.*

## Cosine slots-plus-averaging model

A recent paper [8] suggests that a modified version of the SA model, dubbed cosine slots-plus-average model (cosSA), outperformed the VP model to explain the delay-matching VWM behavior. To enhance the generality of our study, we also followed that work and included this model. Briefly, the cosSA model assumes that the unit memory precision is stimulus-dependent and exhibits a cosine-like periodic fluctuation:

$J_{s}=e^{J_{m}+J_{f}\cos\left(8s\right)}$, (S16)

where $J_{m}$and $J_{f}$ describe the fluctuation of unit memory precision ($J_{s}$) as a function of stimulus *s*. Note that the frequency of the cosine function was derived from the cosine-shaped bias found in our empirical data. We can convert precision $J_{s}$ to the width of von Mises distributions $\kappa_{s}$according to Eq. S4. According to capacity *K*, the discrete memory resource allocation is described as Eq. S11-S14. Moreover, the cosSA model also assumes the response bias is periodic:

$\mu_{s}= \mu_{f}\sin\left(4s\right)$, (S17)

where $\mu_{f}$ adjusts the magnitude of the bias. The probability of a response given the stimulus can be described as:

$p\left(\hat{s}|s\right)=\left\{\begin{matrix} \frac{K}{N}VM\left(\hat{s}|s+\mu_{s},\kappa_{s}\right)+\left(1-\frac{K}{N}\right)\frac{1}{2\pi}, & K\leq N \\ VM\left(\hat{s}|s+\mu_{s},\kappa_{s}\right), & K>N \end{matrix}\right.$ , (S18)

The cosSA model has four free parameters: $J_{m}$,$J_{f}$,$\mu_{f}$ and capacity *K*.

## Equal-precision model

The equal-precision (EP) model is very similar to the VP model, except that an equal amount of resources is assigned to every item and in any trial. Namely, the Eq. S2 does not apply to the EP model. In the EP model, the resource assigned to one item declines as a power function (as Eq. S1). Then the resource at each set size level can be converted to the width of the von Mises distribution using (Eq. S4). The response probability is given by:

, (S19)

where *J_1_* is the resource when set size is 1 (initial resources). The EP model has three free parameters: initial resources $\bar{J}_{1}$, decaying exponent *a*, and choice variability $\kappa_{r}$.

# intuitive model explanations

Despite the mathematical details provided above, we further provide intuitive explanations for each model and highlight their differences based on cartoon illustrations in S1 Fig. Note that all stimuli are 0 because we transformed the reported color to recall errors in each trial.

## Item-limit model

In the IL model (S1A Fig), if the capacity *K* is larger than the set size N (e.g., N=2, K=3, the left panel), all items can enter working memory. The reported color follows a von Mises distribution with the mean as the color of the probed stimulus. If the capacity *K* is smaller than the set size *N* (e.g., N=2, K=3, the right panel), a probed stimulus can be stored within memory with probability *K*/*N* and out of memory with probability (1-*K*/*N*). If the probed stimulus is in memory, the same rule of von Mises distribution applies. If the probed stimulus is out of memory, a subject guesses a color (i.e., with probability 1/2π, the uniform distribution of guessing).

## Mixture model

The mixture model (S1B Fig) shares all components with the IL model. The key difference is that the IL model assumes the same von Mises distribution for both set size levels (i.e., the same width of the blue and the orange distributions in S1A Fig), while the mixture model uses two von Mises distributions with different widths for the two set size levels (i.e., different widths of the blue and the orange distributions in S1B Fig), to compensate the potential different level uncertainty associated with two set size levels. Thus, the mixture model has one additional free parameter than the IL model.

## Slot-plus-averaging and cosine slot-plus-averaging model

The SA model regards memory resources as several discrete chunks (S1C Fig). In the example of Fig 1C, the subject has three (*K* = 3) chunks of resources and the blue cups stand for individual stimulus. If two stimuli are presented (i.e., two cups, set size = 2), the scenario in which the number of resource chunks is larger than the set size, two resource chunks are assigned to one cup and another chunk to the other cup. If the number of resources is smaller than the set size (e.g., four stimuli/cups), one cup will receive no resource, and the subject has to guess if this stimulus/cup is probed. The key difference between the SA model and the three models below is that the SA model assumes discrete resource chunks.

The cosSA model is a modified version of the SA model with three major changes [8]. First, the unit memory precision is stimulus-dependent and follows a periodic function (see Eq. S16 and S1D Fig). Second, it also includes a response bias that is also stimulus-dependent and periodic (see Eq. S17 above and S1D Fig). Third, for simplicity, it does not include the response variability and only includes one uncertainty (i.g., encoding precision) in the processing.

## Equal-precision, variable-precision and variable-precision-with-capacity models.

The EP, VP, and VPcap models share one core assumption: memory resources are continuous, analogous to the amount of juice in a big mug (S1E Fig). A subject assigns the juice (i.e., resources) into different cups (i.e., stimuli). In S1E Fig, the orange cups stand for the mean juice amount an individual cup receives in each set size condition. We can imagine, given the total amount of juice is fixed, the more cups (i.e., larger set size) the less juice on average each cup will receive. This is reflected by the diminishing average amount of juice as set size increases (also see Eq. S1).

Besides the core assumption of continuous resources, the three models have slightly different specifications (S1F Fig). In S1F Fig, all orange cups stand for the mean juice amount in each set size condition, and the blue cups stand for individual stimulus. The EP model assumes that in each set size condition, each cup receives an identical amount of juice (upper row in S1F Fig). In the VP model, however, each cup receives a variable amount of juice even though their average amount is the same as in the EP model. Using two cups as an example, the average amount of juice might be 10 ml but one cup might have 9 ml and the other one has 11 ml. Whether the amount of juice in each cup varies is the key difference between the EP and the VP models. Moreover, both EP and VP models do not constrain the total number of cups. Therefore, a cup will more or less receive a little bit juice even though there is a large number of cups (middle row). In other words, both the EP and the VP models have no concept of capacity. In contrast, the VPcap model not only inherits the assumption of variable precision and but also constrains the maximal number of cups (i.e., capacity *K*) that can receive juice. If the total number of cups (i.e., *N* stimuli) is larger than the capacity *K*, some cups will receive no juice, and the subject has to guess the color of these stimuli.

# Model fitting and comparisons

The BADS optimization toolbox in MATLAB [9] was used to search the best-fit parameters that maximize the likelihood of response data in all trials. BADS has been shown to outperform other default nonlinear optimization algorithms in MATLAB, especially in the problems where gradients on loss function are not available or hard to compute [9]. We fit all models separately in each participant. To avoid local minima, we repeated the optimization process with 20 different initial seeds that are equally spaced within a lower and an upper bound. Parameter bounds were set to be very broad to avoid bias. The parameters with the maximum likelihood value were used as the best-fit parameters for one subject.

We compared the performance of all models fitted in this study. Model comparisons were performed for both groups using both Akaike information criterion (AIC) and Bayesian information criterion (BIC) [10,11] metrics.

**References**

1. van den Berg R, Shin H, Chou W-C, George R, Ma WJ. Variability in encoding precision accounts for visual short-term memory limitations. Proc Natl Acad Sci. 2012;109: 8780–8785. doi:10.1073/pnas.1117465109

2. van den Berg R, Awh E, Ma WJ. Factorial comparison of working memory models. Psychol Rev. 2014;121: 124–149. doi:10.1037/a0035234

3. Bays PM, Husain M. Dynamic Shifts of Limited Working Memory Resources in Human Vision. Science (80- ). 2008;321: 851–854. doi:10.1126/science.1158023

4. Bays PM, Catalao RFG, Husain M. The precision of visual working memory is set by allocation of a shared resource. J Vis. 2009;9: 7.1-11. doi:10.1167/9.10.7

5. Ma WJ, Beck JM, Latham PE, Pouget A. Bayesian inference with probabilistic population codes. Nat Neurosci. 2006;9: 1432–1438. doi:10.1038/nn1790

6. Gold JM, Hahn B, Zhang W, Robinson BM, Kappenman ES, Beck VM, et al. Reduced Capacity but Spared Precision and Maintenance of Working Memory Representations in Schizophrenia. Arch Gen Psychiatry. 2010;67: 570–577. doi:10.1001/archgenpsychiatry.2010.65

7. Zhang W, Luck SJ. Discrete fixed-resolution representations in visual working memory. Nature. 2008;453: 233–235. doi:10.1038/nature06860

8. Pratte MS, Park YE, Rademaker RL, Tong F. Accounting for stimulus-specific variation in precision reveals a discrete capacity limit in visual working memory. J Exp Psychol Hum Percept Perform. 2017;43: 6–17. doi:10.1037/xhp0000302

9. Acerbi L, Ma WJ. Practical Bayesian Optimization for Model Fitting with Bayesian Adaptive Direct Search. Advances in Neural Information Processing Systems 30. 2017. pp. 1836–1846. doi:https://doi.org/10.1101/150052

10. Wit E, van den Heuvel E, Romeijn JW. ’All models are wrong. ’: An introduction to model uncertainty. Stat Neerl. 2012;66: 217–236. doi:10.1111/j.1467-9574.2012.00530.x

11. Burnham KP, Anderson DR. Model Selection and Multimodel Inference: A Practical Information-Theoretic Approach. 2nd ed. Ecological Modelling. New York, NY: Springer-Verlag; 2002.
