# Supplementary material for: Atypically larger variability of resource allocation accounts for visual working memory deficits in schizophrenia
Source: PLoS Comput Biol. 2021 Nov 8;17(11):e1009544. doi: 10.1371/journal.pcbi.1009544 (PMC8601612; doi:10.1371/journal.pcbi.1009544)
Supplement: S2 File — (DOCX) [file pcbi.1009544.s002.docx]

# S2 File: Control experiments 1 to 3

# Control experiment 1: color perception experiment

**Procedure**

To exclude the possibility that the worse VWM performance in SZ was due to worse color perception, we did a control experiment before the main VWM task to measure the color perception ability in the same cohort of subjects. The task is identical to the VWM task except for two modifications. First, only one colored object was shown in the sample array. Second, in the probe array, the colored object appeared again on the screen. A subject needed to choose its color on the color wheel while looking at it. There was 1 block with 50 trials in this task.

**Results**

**Color perception comparison.** We used the circular standard deviation (CSD) of response errors (the circular distance between the original color and chosen color in a trial) to evaluate the performance in the color task. Two participants (one in each group) were excluded from these analyses because they had unreasonably large CSD which were larger than the three-standard deviation threshold. A significant group difference was found (t(117) = -2.470, p = 0.016, d = -0.45), suggesting in general worse color perception in SZ. But this result might also be explained by potential differences in choice variability (e.g., motor control). To exclude the potential confounding of color perception, we further set CSD from the color perception as a co-variate and repeat all statistical analyses (see below).

**VWM performance with color perception as the co-variate.** We added the CSD in the color perception task as a co-variate to the VWM performance comparison of the two groups. The repeated-measure ANCOVA (see the main text for details of variables) results again showed a worse VWM performance at higher set size level (F(1,116) = 54.688, p < 0.001, partial η^2^ = 0.32). The group was also significant (F(1,116) = 7.509, p = 0.007, partial $\eta^{2}$ = 0.061), indicating that HC’s performance was better than SZ’s. The interaction between set size and group was not significant (F(1,116) = 0.062, p = 0.804 partial $\eta^{2}$ = 0.001). Also, the color perception ability had no influence on VWM performance (F(1,116) = 0.849, p = 0.359, partial $\eta^{2}$ = 0.007). These results indicate that the individuals’ color perception thresholds cannot explain the group differences in resource allocation variability seen in the main experiment, indicating that the group differences are indeed memory-related, not due to the general worse color perception in SZ.

**Fitted parameters of the VP model with color perception as the co-variate.** Univariate general linear models were used for comparing fitted parameters between the two groups. We regressed out the factor of color perception by setting. Same as results in the main text, comparable resource decay functions (initial resources, F(1,116) = 0.028, p = 0.867, partial $\eta^{2}$ < 0.001; decaying exponent, F(1,116) = 1.766, p = 0.367, partial $\eta^{2}$ = 0.007) and choice variability (F(1,116) = 1.072, p = 0.303, partial $\eta^{2}$ = 0.009) between SZ and HC were found in this analysis. And SZ showed larger variability in allocating resources (resource allocation variability, F(1,116) = 11.696, p = 0.001, partial $\eta^{2}$ = 0.092). To further rule out the possibility that the higher variability of VWM performance is caused by higher color perception in SZ, we explored the correlations between the CSD in the color perception experiment and the estimated resource allocation variability in the main experiment. We found no significant correlation in both groups (SZ: r = 0.240, p = 0.067; HC: r = -0.041, p = 0.756).

# Control experiment 2: high-set-size color delay-estimation experiment

In our main experiment, in order to recruit a large sample of participants, we reduced the task difficulty and only tested two relatively low set size levels (i.e., 1/3) in order to recruit a large sample of participants. One caveat of this approach is the possible imprecise model fitting because we did not challenge participants’ ceiling performance. It is possible that 1) the VP model might not be the best model, and more importantly, 2) the estimation of the model parameters (e.g., capacity parameter *K* in the VPcap model, see Supplementary Eq.S8) might be inaccurate.

Thus, we repeated the identical color delay-estimation task on another 62 HC subjects (30 females, 19-24 years old). The stimuli and procedure were identical to the task described in the main text except that: 1) the sample array was presented for 200 ms; 2) the set sizes were 2, 4, and 6; 3) there were 100 trials in each set size.

We fitted all seven models to each individual’s data and performed the model comparison. We found that in the total 62 HC subjects, the VP model was the best in 45 and 54 subjects using the AIC and BIC metrics, respectively (see S3 Fig).

# Control experiment 3: orientation delay-estimation experiment

**Procedure**

Data from 26 HC and 9 SZ were obtained in the orientation delay-estimation task (see Supplementary Table 1 for participant information). The data from the HC participants have been previously presented in ref. [1]. SZ were all clinically stable inpatients who met the DSM-IV criteria for schizophrenia[2]. Patients having a history of any other mental or neurological disorders were excluded. All nine patients were receiving second-generation antipsychotic medication. The Positive and Negative Syndrome Scale (PANSS)[3] was used to evaluate the psychotic symptoms of the patients. This scale includes positive symptomatology, negative symptomatology, as well as general psychopathology symptoms. The HC participants were recruited by advertisement. All HC had no current diagnosis of axis 1 or 2 disorders as well as no family history of psychosis nor substance abuse or dependence. All participants are right-handed with normal sight.

Stimuli were presented on a 60 Hz LCD monitor through Matlab psychophysics Toolbox (Version 3). In the sample array, all items were shown on an invisible circle with 10^o^ radius. The length and width of each item in the sample array were 3.7^o^ and 0.6^o^ respectively. Then orientations of the bars were randomly chosen from 1^o^ to 180^o^. The procedure of this task was similar to the color delay-estimation task, except that the sample array was presented for 200 ms (S4A Fig). In the probe array, one of the sample bars appeared as the probe, and the participants were required to adjust the orientation of the probed bar using a computer mouse.

The HC participants completed 5 blocks with 100 trials in each block. The set size of each trial was randomly chosen from 1, 2, 3, 4, 6. The SZ patients complete the experiment in three visit sessions. In each visit, they were asked to finish 10 blocks with 40 trials in each block. The set size levels were 1, 2, 4, 6, and counterbalanced across blocks. We analyzed the behavioral performance using only the data of set size levels 1, 2, 4, 6 because they were the conditions shared by both groups. For model fitting, we included all set size levels (1/2/3/4/6) of the HC group and compared the likelihood, AIC, and BIC per trial to compensate for the trial difference between groups and participants. One patient only finished 9 blocks in the second visit and another patient only finished 8 blocks in the first visit. In total, seven SZ participants completed 300 trials for each set size, one SZ completed 290 trials and one SZ completed 280 trials.

**Results**

**VWM performance of the orientation delay-estimation task.** HC achieved significant lower CSD (F(3, 31) = 62.967, p < 0.001, partial η2 = 0.656) than SZ. Unsurprisingly, both groups performed worse with increasing set size (F(3,31) = 87.682, p < 0.001, partial η2 = 0.895). The group by set size interaction was significant (F(3,31) = 3.043, p = 0.043, partial η2 = 0.228), with smaller group differences on set size 1 and 6, and slightly larger group differences on set size 2 and 4 (p < 0.001 for all pairwise comparisons, Bonferroni corrected).

**Model fitting results.** Similar to the results shown in the main text, we found that among 9 SZ participants, the VP model was the best in 7 participants using AIC and in all 9 participants using BIC. Similarly, in 26 HC participants, the VP model outperformed other models in 21 and 24 participants according to AIC and BIC, respectively.

Resource allocation variability was statistically higher in the SZ participants (t(33) = 5.833, p = 1.576^-6^, Cohen’s d = 2.256), and no significant group differences were detected in other parameters (initial resources, t(33) = 0.437, p = 0.665, Cohen’s d = 0.169; decaying exponent, t(33) = 0.145, p = 0.886, Cohen’s d = 0.056; choice variability, t(33) = 1.651, p = 0.108, Cohen’s d = 0.638).

**Correlation with clinical symptoms.** Resource allocation variability significantly correlated with the severity of general psychopathology symptoms (S6A Fig, r = 0.717, p = 0.03) and negative symptoms (S6B Fig, r = 0.882, p = 0.002), but not positive symptoms (S6C Fig, r = 0.551, p = 0.124).

**References**

1. Zhao Y, Kuai S, Zanto TP, Ku Y. Neural Correlates Underlying the Precision of Visual Working Memory. Neuroscience. 2020;425: 301–311. doi:10.1016/j.neuroscience.2019.11.037

2. American Psychiatric Association. Diagnostic and statistical manual of mental disorders (4th ed.). American Psychiatric Publishing. Washington, DC; 1994.

3. Kay SR, Opler LA, Lindenmayer J-P. The Positive and Negative Syndrome Scale (PANSS): Rationale and Standardisation. Br J Psychiatry. 2018/08/06. 1989;155: 59–65. doi:DOI: 10.1192/S0007125000291514
