# Supplementary material for: Atypically larger variability of resource allocation accounts for visual working memory deficits in schizophrenia
Source: PLoS Comput Biol. 2021 Nov 8;17(11):e1009544. doi: 10.1371/journal.pcbi.1009544 (PMC8601612; doi:10.1371/journal.pcbi.1009544)
Supplement: S1 Table — (DOCX) [file pcbi.1009544.s008.docx]

**S1 Table :** **Demographics and clinical information of the participants in the orientation delay-estimation task**

|  | SZ (N = 9) | |  | HC (N = 26) | |
| --- | --- | --- | --- | --- | --- |
|  | Mean | SD |  | Mean | SD |
| Age | 39.33 | 9.35 |  | 21.31 | 1.94 |
| range | 27-56 | n/a |  | 19-26 | n/a |
| Female/male | 0/9 | n/a |  | 16/26 | n/a |
| Inpatient/outpatient | 9/9 | n/a |  | n/a | n/a |
| Education (years) | 9 | 2.12 |  | 14.12 | 1.63 |
| PANSS - positive | 11.33 | 4.33 |  | n/a | n/a |
| PANSS - negative | 22 | 8.14 |  | n/a | n/a |
| PANSS - general | 31.78 | 14.10 |  | n/a | n/a |

PANSS: ﻿The Positive and Negative Syndrome Scale
